# Excess deaths in China during SARS-CoV-2 viral waves in 2022–2023

**DOI:** 10.1016/j.pmedr.2024.102687

**Published:** 2024-03-15

**Authors:** Prabhat Jha, Patrick E. Brown, Teresa Lam, Ed Morawski, Angus Reid

**Affiliations:** a Centre for Global Health Research, Unity Health Toronto and University of Toronto, Toronto, ON, Canada; b Angus Reid Institute, Vancouver, BC, Canada

## Abstract

**Background:**

The extent to which the Omicron variant of SARS-CoV-2 raised death rates in China during its viral wave of December 2022–January 2023 remains largely undocumented.

**Methods:**

We worked with an established national survey organization to survey 8,004 adults in all 31 administrative areas of China to ask about deaths in families since January 2020. We examined age-specific death rates, focusing on deaths above age 60 years, and at 15–59 years. We compared these to the United Nations (UN) estimates of age-specific mortality in 2019.

**Findings:**

The survey participants were broadly similar to the 2020 census and other national surveys in age, sex, region, and smoking status, but had lower SARS-CoV-2 vaccination rates and higher education levels. There were no differences in reporting of deaths during the Omicron period (after November 2021) versus earlier. The survey captured 456 deaths, of which 329 occurred at ages 60+ years and 212 were of women. At ages 60+ years, death rates approximately doubled during December 2022-January 2023. Deaths at ages 15–59 years did not rise appreciably. The UN estimates approximately 675,000 deaths per month at ages 60+ years in 2019. If rates doubled nationally as in our survey, China had approximately 1.35 million excess deaths from December 2022-January 2023.

**Interpretation:**

China experienced a sharp but short increase in excess deaths among its elderly during the Omicron wave. If death registry data corroborate our estimates of substantial excess deaths in China, the worldwide estimates of excess deaths due to SARS-CoV-2 in 2022–2023 may need upward adjustment.

## 1 Introduction

The People’s Republic of China adopted stringent lockdowns, quarantine, mass testing, and vaccination to combat the SARS-CoV-2 pandemic (Chinese Center for Disease Control and Prevention, 2023, Liu et al., 2021). Despite some early success (Liu et al., 2021, Qi et al., 2022), local outbreaks occurred and a national viral wave of over 100,000 test-confirmed cases ensued in March-April 2022. After abandoning most restrictions and the arrival of the Omicron variants, China reported a sharp increase in cases in December 2022-January 2023 (Chinese Center for Disease Control and Prevention, 2023). Over 100,000 PCR test-confirmed COVID deaths occurred in hospitals during this period (Chinese Center for Disease Control and Prevention, 2023). However, many deaths, even in hospital, went untested or occurred at home.

Excess mortality from all causes is a robust method to capture the direct and indirect mortality from SARS-CoV-2 viral waves (Jha et al., 2022, Msemburi et al., 2023). Direct estimates of excess mortality have not been reported after the original viral wave in Spring 2020 (Liu et al., 2021). We calculated excess mortality in a nationally representative survey of China.

## 2 Methods

### 2.1 Study design and survey

The survey focused on changes in consumption, travel, and family structure during the COVID-19 pandemic, to understand the economic recovery. We developed an online survey with 38 items in February 2023, and pretested it among 200 participants in March 2023, with the final survey implemented in early April 2023. We compared our respondents’ characteristics with Census 2020 or nationally representative surveys on income and health behavior (smoking, SARS-CoV-2 vaccination) (Mathieu et al., 2020, National Bureau of Statistics of China, 2021a, National Bureau of Statistics of China, 2021b, United States Census Bureau, 2024, Yang, 2021, Zhang et al., 2022). Participants took about 8–12 min to complete the survey.

### 2.2 Recruitment and sampling

We worked with an established survey research organization with an online sampling platform. The survey research organization has over two decades of experience in Asia, including in China. The online platform recruits members from mainland China, who receive points used for various merchandise from participation in each survey. Overall internet penetration exceeds 75 % in China (Xinhua, 2023). As of April 2023, the panel covered over three million adults. We provided additional details of the recruitment channels and quality control in Appendix 1.

For this study, the sampling strategy involved creating a profile that was representative of China’s four regions (eastern, central, western, and northeastern) and by age group (18–59 and 60 +) and sex (male, female). Additionally, the survey research organization balanced the sample frame on five tiers that represented over 700 cities, and several income ranges to include lower income participants.

### 2.3 Ethics

The study received ethical approval by the Unity Health Toronto Research Ethics Board (REB # 15-231). Participants in the online panel are recruited voluntarily for various marketing and survey research, and have the right to refuse any particular survey. Those agreeing to this special survey provided online consent.

### 2.4 Survey questions on mortality and outcome variables

We asked all participants “Has anyone left your immediate family since the [Chinese] Lunar New Year [which was on January 25] in 2020?” The possible responses were: 1. “Yes, through divorce or separation,” 2. “Yes, through death,” 3. “No, no one has left my family since the Lunar New Year 2020.” For those responded having any family member who died, we asked “How many people in your immediate family have passed away after Lunar New Year 2020?” Additionally, our participants answered questions about month, year, age and sex for each death that occurred in their families. We constructed a discrete variable tallying the number of deaths in the participants’ families and a dichotomous variable indicating whether any death occurred (1 = any death, 0 = no death). For participants with at least one death in the family since Lunar New Year 2020, another dichotomous variable (1 = since November 2021, 0 = earlier) captured deaths that occurred after the arrival of the Omicron variant.

### 2.5 Analyses

We smoothed the study’s monthly death rates using 3-month rolling averages (although 2-month and 4-month averages showed similar patterns; data not shown). The numerator of the monthly death rates was the number of deaths by age group by month. The denominator was the sum of all family members by age group and month (Appendix 1).

To obtain the level mortality expected in absence of the pandemic, we used China’s population and deaths reported on the World Population Prospects 2022 by the United Nations Population Division to calculate the national age-specific death rates from 2012 to 2019 (United Nations, 2022).We divided the annual rates by 12 to obtain the average monthly rates.

We used logistic regression to determine the likelihood of having a death in the family vs. not, and of having any death in the family since November 2021 vs. any earlier death since Lunar New Year 2020, by region of residence, broad age group, sex, education attainment (primary school, secondary school, some university/vocational school, university, prefer not to answer), monthly household income (5000 RMB or less, 5001 RMB or more, prefer not to answer), smoking history (never, ever), and SARS-CoV-2 vaccination (vaccinated, not vaccinated).

We used Stata/MP 17 to conduct all analyses (StataCorp, 2021). Our data and codebook are available on GitHub: https://github.com/ChinaStudy2023.

## 3 Results

About 210,000 panelists were invited, of whom 8,004 completed the full survey. The response rate of about 4 % is typical of comparable online surveys in China. Because participants reported on the births and deaths of their family members, the survey reached a scope of 35,708 individuals, 6,265 of whom were 60 years or older. The survey captured 456 deaths, 52 of which occurred in Guangdong province, and 28 in Henan and Sichuan provinces each. However, deaths as a proportion of total population among major regions differed little much (143 in eastern, 111 in central and 142 in western regions) except 27 in the northeastern region. Three hundred and twenty-nine deaths occurred at 60+ years and 212 were women. Using the benchmark of 1.1 million deaths in 324 million people before August 2020 (Qi et al., 2022), 105 deaths occurred among the 35,708 survey participants and their families in the same period in our survey. Thus, we had a post-hoc power of 36 % at an alpha of 0.05. To have 80 % power, we would have needed to capture 105 deaths among 101,566 individuals before August 2020.

Our sample was broadly representative of the regional population distribution of China’s 31 administrative regions (Table 1). Compared to the 2020 census and other national surveys, study participants were broadly representative by age, sex, region, and smoking status, but had lower SARS-CoV-2 vaccination rates and higher education levels. Households reporting a death differed little from those that did not, except for more deaths occurring in households with a smoker (Odds ratio or OR = 2.1, 95 % CI 1.5–2.9) and fewer deaths in more educated households (OR = 0.4, Secondary school 95 % CI 0.3–0.6, Some university/Vocational school 95 % CI 0.2–0.7, University 95 % CI 0.2–0.8). There were no differences between reporting of deaths during the Omicron period versus earlier.

**Table 1:** Characteristics of survey participants compared to China’s national census/other data and comparison of households with and without a death.

	Proportions in Census/other surveys	Study number (N) and proportions			Survey-weighted odds ratio (OR) in logistic regression			
Variable		Unweighted N	Weighted %	Weighted % with a death in the family	Any death in family versus (n = 354) versus no deaths (n = 7650)		Deaths in Nov 2021 or after (n = 144) versus deaths earlier (n = 210)	
					OR	(95 % CI)	OR	(95 % CI)
Region ^a^								
Eastern	40 %	3,204	40 %	35 %	Reference		Reference	
Central	26 %	2,048	26 %	26 %	1·2	(0·7 − 2·1)	1·3	(1·1 − 1·7)
Western	27 %	2,179	27 %	33 %	1·4	(1·0 − 2·1)	1·2	(0·6 − 2·4)
Northeastern	7 %	573	7 %	6 %	0·9	(0·4 − 2·0)	2·3	(0·3 − 17·4)

Age group ^b^								
Age 15–59	78 %	7099	86 %	82 %	Reference		Reference	
Age 60 or older	22 %	905	14 %	18 %	1·3	(0·7 − 2·3)	0·8	(0·3 − 2·1)

Sex ^c^								
Male	51 %	4,056	50 %	48 %	Reference		Reference	
Female	49 %	3,945	50 %	52 %	1·4	(1·1 − 1·9)	0·6	(0·3 − 1·1)

Education level ^d^								
Primary school	25 %	299	4 %	10 %	Reference		Reference	
Secondary school	15 %	1,374	18 %	19 %	**0·4**	**(0·3 − 0·6)**	1·1	(0·3 − 4·7)
Some university/Vocational school		2,459	31 %	29 %	**0·4**	**(0·2 − 0·7)**	1·1	(0·2 − 5·3)
University	19 %	3,863	47 %	41 %	**0·4**	**(0·2 − 0·8)**	0·9	(0·2 − 3·6)
Prefer not to answer		9						
Monthly pre-tax household income								
5000 RMB or less		3904	49 %	48 %	Reference		Reference	
5001 RMB or more		4077	51 %	52 %	1·2	(1·0 − 1·4)	0·9	(0·6 − 1·2)
Prefer not to answer		23						

Smoking history ^e^								
Never		5,585	70 %	57 %	Reference		Reference	
Ever	31 %	2,419	30 %	43 %	**2·1**	**(1·5 − 2·9)**	0·7	(0·3 − 1·4)
SARS-CoV-2 vaccinated versus not ^f^	89 %	6,284	78 %	80 %	1·1	(0·6 − 2·0)	0·7	(0·4 − 1·4)
Number of deaths reported in family								
None		7,650	96 %					
One		285	4 %	81 %				
Two or more		51	1 %	19 %				

NOTES: RMB = Renminbi; One respondent who preferred not to describe their sex and two who preferred not to report their education are excluded from the second logistic regression; See references section: a. Bureau of Statistics of China, 2021a; b. United States Census Bureau, 2024; c. Yang, 2021; d. National Bureau of Statistics of China, 2021b; e. Zhang et al., 2022; f. Mathieu et al., 2020.

**Fig. 1:**
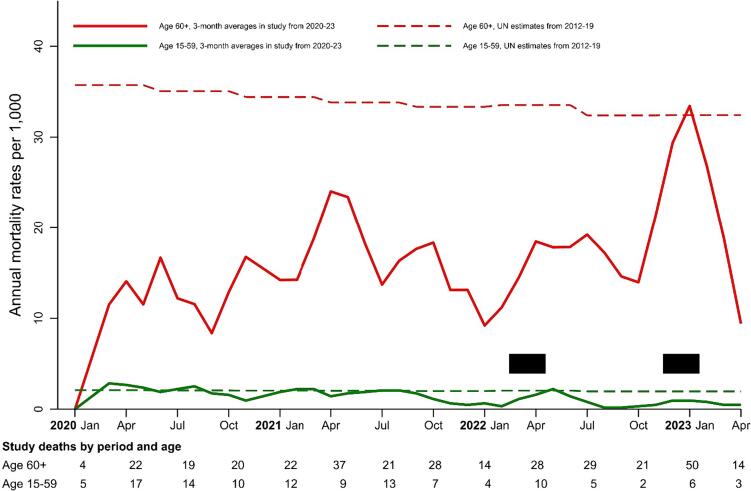
Mortality rates per 1000 at ages 60 years or older and 15–59 years in a nationally representative survey in China from 2020 to 23 compared to United Nations estimates for earlier years. Figure legend: Peak viral periods in March-April 2022 and Dec 2022-Jan 2023 (Chinese Center for Disease Control and Prevention, 2023) are shown in the black rectangles. The numbers of study deaths are shown in the text below the figure. Study deaths use rolling three-month averages. The annual mortality rates are per 1,000 person-years. The smoothed UN estimated deaths are for 2012–19 (https://population.un.org/wpp/).

At 60+ years, the average death rates per 1000 fluctuated but approximately doubled during December 2022-January 2023, showing 50 deaths as against about 24 expected (Fig. 1). Survey death rates at these ages were lower than the UN death rates, which are more complete as they draw upon death registration and census data. Death rates at ages 15–59 showed less fluctuation and were closer to the UN death rates.

Using the UN estimates, China had about 8·1 million deaths at ages 60+ in 2019 (Xinhua, 2023), which (ignoring seasonality) equates to approximately 675,000 deaths monthly. If death rates doubled nationally during December 2022-January 2023, as they clearly did so in our survey, mainland China had 1·35 million excess deaths at 60+ years.

## 4 Discussion

Our estimate of 1·35 million excess deaths in China’s last SARS-CoV-2 wave of December 2022-January 2023 is crude by necessity, but consistent with results drawn on modelling-based increases in cremations (Dyer, 2023) and death registration data in China’s special administrative regions. Hong Kong saw about 70 % excess deaths in March-April 2022 (when the increase in cases was smaller than in December 2022-January 2023) and Macau saw about 170 % excess deaths during the later peak (Karlinsky and Kobak, 2021). Both regions have better health systems and reporting than the whole of China. Although our estimate of excess deaths is lower than the 1.9 million based on internet searches for funerals (Xiao et al., 2023), both estimates above 1 million are an order of magnitude larger than official totals (which focus only on PCR-confirmed deaths in hospital). China's National Bureau of Statistics reports 10.4 million deaths in 2022 and 11.1 million in 2023, compared to an average of 10.0 million annually from 2018-2020 (National Bureau of Statistics of China, 2023). The additional 1.1 million death total in 2023 is consistent with our estimate. Excess deaths were concentrated at ages 60+ also during the original wave of spring 2020 (Liu et al., 2021), although we captured too few deaths before August 2020 (Qi et al., 2022) to examine 2020 patterns. Additionally, China has high overall vaccination coverage of 90 %, but its elderly are disproportionally less fully (3-doses) vaccinated (Davidson, 2022).

We did not observe over-reporting of deaths after arrival of the Omicron variant in November 2021 versus earlier. Indeed, we sequenced questions to avoid such spurious reporting. Nevertheless, sample surveys generally underestimate deaths (Keyes et al., 2018) and are a poor substitute for timely release of China’s high-quality death registration data. Although our estimate of more than one million excess deaths is much larger than China’s 122,000 deaths from COVID-19 (World Health Organization, 2023), our estimates include deaths of any cause. Furthermore, China’s 2025 Census and its Disease Surveillance Point system (covering 20 % of the population) could each quantify excess deaths directly by reporting deaths from January 2020 onward by location, age, sex, and date.

Despite an older population, China's excess deaths during the Omicron wave of December 2022-January 2023 were about half of what India experienced during its very large Delta wave of April-May 2021 (Jha et al., 2022, Msemburi et al., 2023). If indeed China had substantial excess deaths in late 2022/early 2023, they would suggest a significant upward revision of worldwide excess deaths from SARS-CoV-2 infection through mid-2023 from the estimated 13–17 million deaths as of December 2021 (Msemburi et al., 2023).

## 5 Role of the funding source

The study is funded by the 10.13039/501100000024Canadian Institutes of Health Research (FDN 154277). The funder had no role in data collection, analysis, interpretation of the findings, writing of the paper, or the decision to submit.

## Funding

Canadian Institutes of Health Research.

## CRediT authorship contribution statement

**Prabhat Jha:** Conceptualization, Formal analysis, Methodology, Supervision, Writing – original draft, Writing – review & editing. **Patrick E. Brown:** Formal analysis. **Teresa Lam:** Data curation, Formal analysis. **Ed Morawski:** Conceptualization, Project administration. **Angus Reid:** Conceptualization.

## Declaration of competing interest

The authors declare that they have no known competing financial interests or personal relationships that could have appeared to influence the work reported in this paper.

## Data Availability

We have shared the link to our data and data dictionary in the manuscript.
